# Helminth Infection of the Loggerhead Sea Turtle *Caretta caretta* along the Coasts of Sicily and the North West Adriatic Sea

**DOI:** 10.3390/ani11051408

**Published:** 2021-05-14

**Authors:** Antonino Gentile, Tullia Amato, Andrea Gustinelli, Maria Letizia Fioravanti, Delia Gambino, Vincenzo Randazzo, Giulia Caracappa, Domenico Vicari, Marco Arculeo

**Affiliations:** 1 Istituto Zooprofilattico Sperimentale della Sicilia “A. Mirri”, Via Marinuzzi 3, 90100 Palermo, Italy; deliagamb@gmail.com (D.G.); vincenzorandazzo78@gmail.com (V.R.); giulia.caracappa@gmail.com (G.C.); domenico.vicari@izssicilia.it (D.V.); 2 Department of Veterinary Medical Sciences, University of Bologna, Via Tolara di Sopra 50, 40064 Ozzano dell’Emilia, Italy; tullia.amato2@unibo.it (T.A.); andrea.gustinelli2@unibo.it (A.G.); marialeti.fioravanti@unibo.it (M.L.F.); 3 Department STEBICEF, University of Palermo, Via Archirafi 18, 90123 Palermo, Italy; marco.arculeo@unipa.it

**Keywords:** trematoda, nematoda, loggerhead sea turtle, *Caretta caretta*, Mediterranean sea

## Abstract

**Simple Summary:**

We report new data on the presence of trematode and nematode parasites species in stranded specimens of loggerhead sea turtles (*Caretta caretta*). These parasites can potentially cause severe damage to internal organs, endangering the lives of the animals. The results showed a greater presence of digeneans.

**Abstract:**

We provide new data on the presence of helminth parasites in 64 individual loggerhead sea turtles *Caretta caretta* stranded along the coasts of Sicily and the northwest Adriatic Sea between June 2014 and August 2016. The necropsy examination revealed 31 individuals (48.4%) positive for endoparasites, showing a greater prevalence of trematodes than nematodes. In particular, seven species and a single genus of Trematoda (*Hapalotrema*) and a single species and genus of Nematoda (*Kathlania*) were identified. Among the Digenea flukes the species with the highest prevalence of infection were *Rhytidodes gelatinosus* (34.6%) and *Hapalotrema* sp. (33.3%), while among the Nematoda they were *Kathlania* sp. (33.3%) and *Sulcascaris sulcata* (33.3%). Analysis of variance (ANOVA) was applied among the recovery sites of the stranded loggerhead sea turtles and prevalence of endoparasites was used to highlight any relationship between the parasites and the origin of the hosts. ANOVA showed significant differences (*p* < 0.001) among the data used.

## 1. Introduction

The loggerhead sea turtle *Caretta caretta* has a cosmopolitan distribution and is the most common sea turtle in the Mediterranean Sea. The survival of the species, however, is highly endangered throughout the Mediterranean Sea due to the continuous increase in anthropization of the coasts. In fact, pollution by chemical substances, plastic (micro and macro) debris [1], reduction of nesting sites, collisions with boats and accidental catches by fishing gear [2] are all factors that jeopardize the survival of sea turtles. To the factors that afflict this species we must add those related to the presence in their internal organs and in some parts of the body surface of species of parasites that can cause health problems, including death [3,4]. Moreover, according to Irvine [5] and Preston and Johnson [6] the role of ecto- and endoparasites can be considered among the main factors that can compromise the biology of the host and consequently also the survival of entire populations. An example of an ectoparasite that can cause important pathologies in *C. caretta* are the leeches, that can act as a vector of the fibropapilloma virus [7].

The endoparasite fauna of sea turtles is diverse, mainly comprising protozoa and helminths [8], the latter representing undoubtedly the most common endoparasite taxa, among which trematodes are the most frequently reported. Digenean flukes require hosts to complete their life cycle [9] and sea turtles are definitive hosts of the adult parasites, localized in the alimentary tract, in the bile ducts or more rarely in the urinary bladder and vascular system. Among the parasites with the greatest pathogenic potential bloodflukes of the Spirorchiidae family whose infections have in some cases been related to mortality episodes [10,11]. The pathogenic effect of the adult stage of these parasites is closely related to their localization, mainly the circulatory system, and to the severe inflammatory reactions caused in various organs and tissues by their eggs eliminated through the bloodstream. Cases of Spirorchidiasis have been recorded in different parts of the world and in particular in the north-western and central Atlantic Ocean [11,12,13], in the Indian Ocean and in the north-eastern Pacific Ocean [14,15,16], while in the Mediterranean Sea infections by Spirorchiidae have been reported by Monticelli [17], Marchiori et al. [18] and Santoro et al. [10].

Over the past 10 years several studies have been conducted on helminths present in stranded and free-living individuals of *C. caretta* in different areas of the Mediterranean Sea [19,20]. Most of these studies refer mainly to the Central Tyrrhenian Sea, Adriatic and Ionian Sea [10,19,20,21], while knowledge about the Sicilian coasts is still very limited [20].

This study provides new information on the endohelminth species found in loggerhead sea turtles stranded along the Sicilian and Adriatic coasts.

## 2. Materials and Methods

### 2.1. Sample Collection

**Figure 1:**
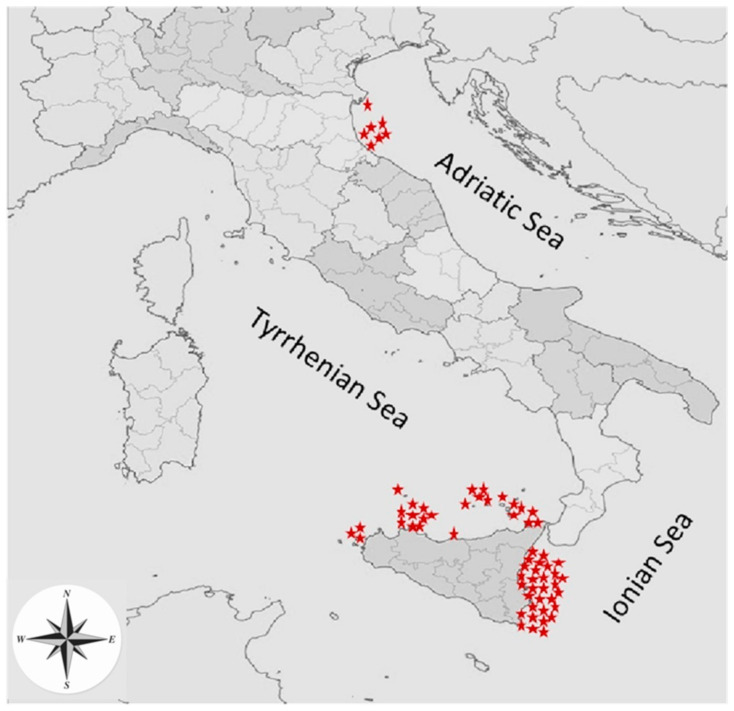
Specimen origin of *C. caretta* along Sicily and Adriatic coasts.

During the period of June 2014–August 2016, sixty-four specimens of stranded loggerhead sea turtles were examined. Among these 31 individuals came from the Ionian Sea, 26 from Tyrrhenian Sea and 7 from the coasts of Ravenna, northern Adriatic Sea (Figure 1). 

Fifteen specimens were recovered already dead, while the remaining 49 were recovered stranded alive. Stranded individuals were rescued by qualified personnel of the Regional Center for the Recovery of Sea Turtles of the “Istituto Zooprofilattico Sperimentale della Sicilia A. Mirri” (IZSSi), and of the Istituto Zooprofilattico Sperimentale della Lombardia e dell’Emilia Romagna (IZSLER), to perform surgery and/or rehabilitation treatments. Individuals were, whenever possible, sexed (through the vision of the gonads during necropsy; while in alive individuals the total length of the tail and post cloacal was measured) and the curved carapace length (CCL), width (CCW) and weight were measured.

### 2.2. Parasitological Examination

Parasitological examination was conducted in all internal organs of dead specimens, whereas feces were analyzed in alive and dead animals. During the necropsy, the internal organs and the entire gastrointestinal tract were analyzed, following the suggestions of Flint et al. [22]. According to Flint et al. [22] the gastrointestinal tract was filled with water, shaken manually and the water collected was subsequently filtered through sieves with decreasing diameter pores (500–212 μm) to collect parasites and eggs. A centrifugal sedimentation/flotation technique using a high-density solution (sodium nitrate and glucose of density 1300/1350) was used to search for helminths eggs in faecal material [18]. Parasites isolated from the sample were washed in saline solution and, after a preliminary gross identification, fixed in 5% formalin for preservation [23]. Subsequently, parasites and eggs were observed under an optical microscope, and identified according to the taxonomic keys proposed by Greiner et al. [24], Gagno [25], Blair and Limpus [26], Bray [27] and Gibson et al. [28].

### 2.3. Statistical Analyses

Prevalence of parasites infection and the 95% confidence intervals (CI) were calculated using the Quantitative Parasitology Program [29]. One way ANOVA tests were used to determine the statistical significance among the origin of the hosts (Tyrrhenian, Ionian and Adriatic Sea) and their parasites by the statistical software MINITAB 17 (University Park, PA, USA).

## 3. Results

Sixty-four individuals of stranded loggerhead sea turtle *C. caretta* from Ionian Sea (*n* = 31), Tyrrhenian Sea (*n* = 26) and Adriatic Sea (*n* = 7) were analyzed. The carapace length (CCL) for the most part of the specimens was between 41–69 cm (*n* = 45), while those with CCL > 70 cm and <40 cm were respectively n. 4 and n. 15. The sex was determined only on 32 individuals (24 females and eight males), showing an unbalanced gender ratio in favor of females.

All the individuals analyzed in necropsy did not show macroscopic lesions referable to parasitic infections. Small focal lesions were found in the myocardium and urinary bladder mucosa, although in no case any parasite was isolated from these lesions. Evidence of parasitic infection was detected in some individuals by findings of eggs belonging to digenean flukes and nematodes. 

The parasitological analysis revealed the presence of parasites in 12/31 individuals from the Ionian Sea (38.7%), 13/26 individuals from the Tyrrhenian Sea (55%) and 6/7 individuals from the Adriatic Sea (85.7%). The overall prevalence of infection was 48.4% (Table 1).

With respect to the 64 individuals stranded along the Sicilian coasts (*n =* 57) and the Adriatic coast (*n =* 7), the coprological analysis highlighted the almost exclusive presence of digenean flukes. Nematodes of the genus *Kathlania* sp. were found in a single individual from the Ionian Sea and in two individuals from the Adriatic Sea, while the species *S. sulcata* with its eggs was found only in two individuals from the Adriatic Sea (Table 1).

In total, eight species of Trematoda Digenea and two species of Nematoda have been found (Table 1). It must be added that one specimen was generically ascribed to Digenea and another to Nematoda, since it was not possible to determine the lower taxa due to the inadequate preservation of the parasites. Three of the 10 taxa identified (excluding those generically indicated Nematoda and Digenea) are shared between the three sites of recovery of the animals, while two taxa are exclusive to the Adriatic Sea and two shared by the Tyrrhenian and Ionian Sea. It was interesting the finding in two individuals recovered on the Adriatic shores of some eggs of Spirorchiidae of the genus *Hapalotrema*, in one case within brownish focal lesions in the intestinal mucosa.

The highest prevalence values of infection were showed for *Rhytidodes gelatinosus* in specimens from the Tyrrhenian Sea (34.6%), from the Adriatic Sea (33.3%), and for *Enodiotrema megachondrus*, with values ranging between 9.7% in specimens from the Ionian Sea and 16.7% from the Adriatic Sea. The prevalence values of infections reported in Table 1 show a certain heterogeneity among the three sites examined. This is confirmed by the One way ANOVA test carried out taking into account the three sites of recovery of the animals which was highly significant (*p* < 0.001, Table 2).

## 4. Discussion

Data provided in the present study contribute to increase the knowledge on the presence of parasitic infection by digenean trematodes and nematodes in individuals of loggerhead sea turtle stranded along the Sicilian and the North Adriatic coasts. The ANOVA analysis between the three sites (Tyrrhenian, Ionian and Adriatic Sea) confirmed a significant difference among them (*p* < 0.001). According to Gracan et al. [21] and Valente et al. [30] this difference could be attributed to the feeding ecology or habitat preferences of *C. caretta*; in fact, authors found [21,30] a different composition of the helminthic fauna during necropsies of individuals of *C. caretta* from different parts of the Mediterranean Sea and the Atlantic Ocean. This difference is also evidenced by the exclusive presence of the nematode *S. sulcata* in the individuals from the Adriatic Sea, previously reported by Manfredi [31], Scaravelli [32], Gracan [21], Santoro [33] and by the presence of the eggs of digenean Spirorchiidae, *Hapalotrema* sp. [4]. 

The most important aspect of these results is the evidence of Spirorchiidae and nematode infections in *C. caretta* in the Mediterranean Sea [33,34] with particular reference to the Sicilian coasts where reports on the latter parasites are very few. According to Santoro et al. [3,33], who studied the infection prevalence of Spirorchiidae flukes *Hapalotrema mistroides* and the Nematoda *S. sulcata* in loggerhead sea turtles from different areas of the Mediterranean Sea, the prevalence of these highly pathogenic parasites could be linked to the different habitats frequented by the hosts. In fact, during their life cycle *C. caretta* frequent different habitats showing differences in the diet composition depending on the area where they feed [35]. Moreover, the intermediate hosts of these parasites are mainly bivalve molluscs and gastropods of which the Adriatic Sea is quite rich [36,37,38]. The natural presence in the Adriatic Sea of bivalve molluscs such as mussels, is increased by the various mussel farm ropes present in the northernmost part of the Adriatic Sea. Santoro et al. [3,32] have recently shown that the high prevalence of *S. sulcata* in the Gulf of Naples [3] and in the Adriatic Sea [33] in loggerhead sea turtles is related to the consumption of mussels or other species of bivalves. In fact, while for the *Caretta caretta* samples from the Gulf of Naples they found a correlation between the consumption of mussels, including farmed ones, and the presence of *S. sulcata*, this close correlation was not highlighted in the *C. caretta* samples analyzed from the Adriatic Sea although this does not mean that *S sulcata* is not present. Most likely the presence of *S. sulcata* in *C. caretta* is attributable to the consumption of bivalve molluscs, mainly Pectinidae, as demonstrated by Lazar et al. [35]. This observation was recently confirmed by Marcer et al. [39] in which a correlation between the presence of *S. sulcata* in the Adriatic loggerheread sea turtles and the consumption of the intermediate hosts such as the bivalves of the Pectinidae family is highlighted. 

These data show that in the Adriatic Sea *S. sulcata* reach the loggerhead sea turtles through the consumption of its intermediate host such as *P. jacobeus* and *A. opercularis*, while, although evidenced in a few samples (Santoro et al., [3], *S. sulcata* can also be found in the mussels collected in the Tyrrhenian Sea. In fact, the presence of *S. sulcata* had been described in clams and scallops in Pretto et al. [40] and Marcer et al. [39] and according to Lazar et al. [35] they represent the most frequent ingested bivalve molluscs for the Adriatic *C. caretta*. Conversely to Santoro et al. [33] who examined some specimens of loggerhead sea turtle from the Sicilian coasts, in our samples we did not find any individual with these parasites. This apparent contrast could be explained by the scarcity of these molluscs (scallops, clams and mussels) in Sicily. We know that *C. caretta* is a carnivorous generalist species, also opportunist, and can use different trophic resources based on their availability and their trophic phases [35]. Furthermore, according to Gracan et al. [21], the difference in the structure of the endohelminth community can be explained through the ontogenesis and life history of the host and the parasite.

The same arguments could be made for Spirorchiidae flukes *Hapalotrema mistroides,* found exclusively in Adriatic samples, although, unlike *S. sulcata,* these blood flukes showed a complex life cycle involving up to two hosts [41]. In fact, as pointed out by Chapman et al. [41], to date very little is known about the intermediate hosts of marine blood flukes, even if it is known that blood fluke infection is by direct penetration of the cercaria into the sea turtles [42]. As previously reported for bivalve molluscs, the Adriatic Sea has, compared to the Sicilian coasts, also greater quantities of gastropod molluscs which are part of the diet of the Adriatic loggerhead sea turtles [21,35]. This would explain the presence of both parasites exclusively in our Adriatic samples.

Our data compared with those reported in literature confirmed that some differences in the helminthofauna of stranded loggerhead sea turtles are connected with the recovery sites of the specimens. In future, the endohelminthic fauna of a loggerhead sea turtle could be used, as in the case of fish, as a biological marker of its origin or permanence in different foraging areas [43]. The possible identification of foraging areas can also be supported by advanced technologies such as the use of satellites and drones [44]. Mingozzi et al. [44] using satellite tracking demonstrated that several females, that laid their eggs along the Ionian coast of Calabria, headed to foraging areas along the coasts of Tunisia, highlighting a strong individual fidelity to their foraging areas.

In conclusion, this study improves data on the helminthofauna of *C. caretta* and in particular on the incidence of spirorchiids infection, highlighting the scarce knowledge on its life cycle and on its intermediate hosts. The potential negative influence of the helminthofauna on the demography and health status of the loggerhead sea turtles requires further studies. 

## Figures and Tables

**Table 1:** Presence of parasites and prevalence in the analyzed samples. In parentheses 95% Confidence Interval.

Site	Parasites	Positive Sample	Prevalence (%)
Ionian Sea (*n* = 31)	Trematoda Digenea		
	*Calycodes anthos*	1	3.2 (0.1–16.7)
	*Cymatocarpus undulatus*	2	6.4 (0.8–21.4)
	*Enodiotrema megachondrus*	3	9.7 (2–25.8)
	*Orchidasma amphiorchis*	1	3.2 (0.1–16.7)
	*Pronocephalus obliquus*	1	3.2 (0.1–16.7)
	*Rhytidodes gelatinosus*	2	6.4 (0.8–21.4)
	*Sthyphlotrema solitarium*	3	9.7 (2–25.8)
	Nematoda		
	*Kathlania* sp.	1	3.2 (0.1–16.7)
Tyrrhenian Sea (*n* = 26)	Trematoda Digenea		
	*Calycodes anthos*	2	7.7 (0.9–25.1)
	*Cymatocarpus undulatus*	3	11.5 (2.4–30.2)
	*Enodiotrema megachondrus*	4	15.4 (4.4–34.9)
	*Orchidasma amphiorchis*	2	7.7 (0.9–25.1)
	*Rhytidodes gelatinosus*	9	34.6 (17.2–55.7)
Adriatic Sea (*n* = 6)	Trematoda Digenea		
	*Enodiotrema megachondrus*	1	16.7 (0.4–64.1)
	*Hapalotrema* sp.	2	33.3 (4.3–77.7)
	*Orchidasma amphiorchis*	1	16.7 (0.4–64.1)
	*Rhytidodes gelatinosus*	2	33.3 (4.3–77.7)
	Unidentified digenea	1	16.7 (0.4–64.1)
	Nematoda		
	*Kathlania* sp.	2	33.3 (4.3–77.7)
	*Sulcascaris sulcata*	2	33.3 (4.3–77.7)
	Unidentified nematode	1	16.7 (0.4–64.1)

**Table 2:** One-way ANOVA based on the samples abundances in the three different recovery sites of the hosts (Tyrrhenian, Ionian and Adriatic Sea). Significance level α = 0.05. DF, degree of freedom; Adj SS, adjusted sum of squares; Adj MS, adjusted mean of squares; F-value, F statistic, StDev standar deviation.

**Source**	**DF**	**Adj SS**	**Adj MS**	***F*** **-Value**	***p*** **-Value**
Location	2	27.60	13.7976 0.8187	16.85	0.000
Error	18	14.74			
Total	20	42.33			
Means					
**Sample Site**	***N*** **of Taxa**	**Mean**	**StDev**	**95% CI**	
Adriatic	8	4.929	0.900	(4.257, 5.601)	
Ionian	8	2.306	0.595	(1.633, 2.978)	
Tyrrhenian	5	3.755	1.283	(2.905, 4.605)	

## Data Availability

The data presented in this study are available on request from the corresponding author.
